# A framework for concepts of reserve and resilience in aging

**DOI:** 10.1002/alz.087228

**Published:** 2025-01-09

**Authors:** Yaakov Stern

**Affiliations:** ^1^ Columbia University Irving Medical Center, New York, NY USA

## Abstract

**Background:**

This talk will review the Framework and discuss how it can be used to help conceive of and design research studies into cognitive reserve, brain maintenance and brain reserve. It will also highlight several funded pilot awards that emerged to implement the operational definitions. Finally, the talk will highlight the fourth Collaboratory workshop at which a set of collaborative research projects were initiated that make use of the Framework and address both human and nonhuman cognition.

**Method:**

The Framework offers consensus operational definitions for three aspects of an overall concept of “resilience:” cognitive reserve, brain maintenance and brain reserve.

**Result:**

Cognitive reserve is defined as a property of the brain that allows for cognitive performance that is better than expected given the degree of life‐course related brain changes and brain injury or disease. Brain maintenance was defined as the relative absence of changes in neural resources or neuropathologic change over time as a determinant of preserved cognition in older age. Finally, we define the concept of brain reserve to reflect the neurobiological status of the brain at any point in time. The Collaboratory funded 12 pilot awards that utilize these definitions. These awards are summarized in the supplement to the published Framework. At a fourth Collaboratory meeting, investigators gathered to initiate new collaborative research based on the framework. These include several projects that will be discussed at this talk. Additional projects will be described including evaluation of the interaction of physical activity and stress on cognitive performance, sociocultural factors and cognitive resilience, the contribution of molecular measures such as astrocytes and glia to cognitive reserve.

**Conclusion:**

The framework defines three concepts developed to guide research. These operational definitions are also of use if researchers choose to use alternate names for concepts. The use of a common vocabulary and operational definitions will facilitate even greater progress in understanding the factors that are associated with successful aging. The studies we discuss demonstrate that the Framework can underline a broad range of human and nonhuman studies.

